# Risk assessment of healthcare workers’ exposure to physical load in relation to patient handling and movement: a feasibility study of the instrument TilThermometer

**DOI:** 10.1186/s12891-024-07508-9

**Published:** 2024-05-21

**Authors:** Charlotte Wåhlin, Sebastian Buck, Paul Enthoven, Maria Andreassen, Jan Sandqvist, Patrik Haraldsson, Jenni Fock, Emma Nilsing Strid

**Affiliations:** 1 Occupational and Environmental Medicine Centre, Department of Health, Medicine and Caring Sciences, Division of Prevention, Rehabilitation and Community Medicine, Unit of Clinical Medicine, Linköping University, Linköping, Sweden; 2 Unit of Intervention and Implementation Research for Worker Health, Institute for Environmental Medicine, Karolinska Institutet, Stockholm, Sweden; 3 Department of Health, Medicine and Caring Sciences, Division of Prevention, Rehabilitation and Community Medicine, Unit of Physiotherapy, Linköping University, Linköping, Sweden; 4 Department of Health, Medicine and Caring Sciences, Division of Prevention, Rehabilitation and Community Medicine, Unit of Occupational therapy, Linköping University, Norrköping, Sweden; 5 Occupational Safety and Health Care, Region Jönköping County, Jönköping, Sweden; 6 School of Health and Welfare, Academy for Improvement of Health and Welfare, Jönköping University, JönköpingJönköping, Sweden; 7 Unit of Strategic Development, Linköping University Hospital, Linkoping, Region Östergötland Sweden; 8 Faculty of Medicine and Health, University Health Care Research Center, Örebro University, Örebro, Sweden

**Keywords:** Occupational health, Ergonomics, Nursing, Risk assessment, Health personnel workers, Safety management, Working conditions environment

## Abstract

**Background:**

Work-related musculoskeletal disorders are common among healthcare workers (HCWs) but might be prevented by risk assessment and further promotion of occupational safety and health.

The aim of this study was to investigate if the risk assessment instrument TilThermometer can be used to identify risk profiles of physical exposure in HCWs working with patient handling and movement (PHM). Further aims were to describe HCWs’ perceptions and experiences of using the TilThermometer.

**Methods:**

This feasibility study has a mixed design methodology. In total, 54 HCWs from 17 Swedish care units participated and performed risk assessments with the TilThermometer. Data collected from the risk assessments were used to identify risk profiles of physical exposure. HCWs’ experiences of using the TilThermometer were collected from activity logs and analysed qualitatively. Three questionnaires were used to assess perceived acceptability, appropriateness, and feasibility of the risk assessment, and eight study specific questions were used for perceived usefulness.

**Results:**

The TilThermometer was used at the care units by assessing each care recipient, and when compiling the data at a group level, a summarized risk profile for the care unit could be provided. Risk for physical exposure was reported as high in two work tasks; no care unit used the high-low adjustable seat when showering care recipients sitting down, and 13% used the recommended assistive devices when putting compression stockings on. However, 99% used high-low adjustable assistive devices when caring and bathing care recipients lying down. TilThermometer was described as easy to use, enabling team reflections and providing an overview of the care units’ recipients and workload, but difficulties in categorizing for mobility groups were also reported. The TilThermometer was, on a five-point scale, perceived as acceptable (mean 3.93), appropriate (mean 3.9), and feasible (mean 3.97). These scores are in line with questions evaluating usefulness.

**Conclusion:**

The risk assessment provided risk profiles with potential to contribute to care units’ development of a safe patient handling and movement practice. The findings suggest that the TilThermometer can be used to assess risks for physical exposure in relation to patient handling and movement in care units at hospital and nursing homes.

## Introduction

There is a relationship between working conditions and work-related musculoskeletal disorders (WMSDs) among healthcare workers (HCWs) [1–4]. The WMSDs are primarily caused by the performance of work tasks and they encompass a range of soft-tissue injuries affecting muscles, nerves, tendons, ligaments, joints, cartilage and bones in the upper and lower limbs, neck, and lower back resulting in disability and pain, whether localised or widespread [5, 6]. Primarily nurses, but also other HCWs are affected by WMSDs [3, 7–10]. The causes of WMSDs are multifactorial, including aspects of the work environment that involve physical workload, organizational and psychosocial factors, as well as individual factors [1, 4, 8, 11]. The interaction between these work environmental factors and individual factors must be taken in consideration in order to promote safety and to prevent injuries at the workplace [7, 8]. Musculoskeletal injuries often occur in work tasks such as carrying, lifting or moving heavy material or equipment, but also in caring situations when HCWs are working with patient handling and movement (PHM) [2, 8]. PHM includes work tasks such as lifting, transferring, and repositioning of care recipients, as well as awkward and static postures and repetitive movements [2, 4, 8, 9]. Previous research has shown that repetition of awkward postures and high frequency of performing PHM is associated with increased risk for WMSDs such as back injury [7, 11]. Lack of support and poor collaboration between colleagues further increase that risk [11]. These findings indicate that to prevent WMSDs among HCWs, there is a need to assess risks and to select relevant work interventions to ensure a safe work environment. For example, the use of assistive devices can decrease the physical load and reduce the risk of WMSDs [11–14]. Previous research suggests that both an individual and organizational strategy is needed to promote the use of assistive devices [15]. In Sweden, as in many countries, it is mandatory for managers to regularly work with systematic work environment management, perform risk assessments to eliminate, control, and reduce those risks, and promote a culture of safety [16]. From the perspective of occupational safety and health, it is suggested that identification of risks and taking appropriate measures in a timely manner promotes better working conditions and improves occupational safety, job satisfaction, and commitment throughout the organization [17]. A system perspective is needed to ensure safety for both care recipients and HCWs, as presented in the model of System Engineering initiative for patient safety [18]. According to Hignett et al. [19], assessments of risks are necessary in healthcare to build a safety structure and safety culture. It is central to understand the exposures in relation to specific work tasks that HCWs perform in their daily caring work and the influence of different work environment factors. Intensity, duration, and frequency of work tasks are of great importance when analyzing physical exposure during PHM, and can be assessed by different risk assessment instruments [20]. The findings from a recent review by Kugler et al. [21] indicates a potential positive impact on musculoskeletal injury rates by interventions incorporating risk assessment, but the evidence is of very low certainty and further research is needed. The TilThermometer is one of the presented risk assessment instruments that can be used in healthcare workplaces to assess the risk of physical exposure [20]. In the TilThermometer, the HCWs' risk for physical exposure is assessed in different types of PHM situation in relation to the care recipients’ function and level of activity [22, 23]. The Dutch version of the TilThermometer was developed in 2002 as a web-based tool, and is free and available in Swedish and several other languages [24]. The TilThermometer has recently been culturally adapted and translated from Dutch into a Swedish version [25]. However, the Swedish version of the instrument has not been tested and scientifically evaluated for practical use at care units. In Sweden, healthcare is managed by either the regional authority or the local authority (or municipality) and is, to a large extent, tax funded. Care units are present both within municipal settings and within hospital care facilities. It is of particular interest to compare these two types of care units due to their differing organizational structures, care recipients, demographics, and varying levels of resources and support. By evaluating the similarities and differences in the use of the TilThermometer within these contexts, we can gain insights into its applicability across diverse healthcare settings. Additionally, exploring the responses from HCWs within both hospital care units and nursing home care units allows for a comprehensive understanding of the potential impact and effectiveness of implementing the TilThermometer in various healthcare environments. In the present study, the TilThermometer will be evaluated due to its potential of mapping the severity of the care recipients, exploring physical exposure, create risk profiles, providing an overview of physical exposure on a care unit level and facility level in relation to PHM. Inquiry into HCWs’ experiences and perceptions of using the TilThermometer for assessing risk of physical exposure would inform the development and implementation of interventions for safe PHM incorporating the instrument. The findings may contribute with knowledge on how to promote occupational safety and health in hospital and nursing home care units.

### Aims

The aim was to investigate if the TilThermometer can be used to assess risks for exposure to physical load in HCWs when working with PHM in a Swedish context, and whether the TilThermometer can be used to provide risk profiles for physical exposure at care units and identify HCWs’ use of available assistive devices. Other aims were to describe HCWs’ experiences of using the TilThermometer and to describe HCWs’ perceptions of the acceptability, appropriateness, feasibility, and usefulness of the TilThermometer. Finally, the results from care units in hospitals and nursing homes will be compared.

## Methods

### Design

This is a feasibility study with a descriptive cross-sectional design in which both quantitative and qualitative data were collected. Feasibility studies are designed to answer an overarching question: Can it work? [26]. A feasibility study was conducted to inform a large cluster randomized controlled trial (RCT) implementing and evaluating risk assessment and strategies for safe PHM. The focus of this study is on the feasibility objectives of intervention acceptability and suitability, ability to implement the intervention, and preliminary evaluation of participant responses to the intervention [26].

### Sample and recruitment

The study was performed at Swedish care units, including hospital care units (regional healthcare sector) and nursing homes (municipal healthcare sector). The care units were located in the counties of Östergötland, Jönköping and Västernorrland. Exclusion criteria for healthcare units were outpatient home nursing, paediatric care, parts of emergency care, and psychiatric care. We recruited a convenience sample of care units through networks within healthcare, nursing homes and occupational health services. Some members of the research project management (CW; JF; SB; MA; PH) held meetings at the care unit or digitally to provide information about the study aim and procedures to interested care units (June to August 2021). The care unit’s manager, some HCWs, and safety representatives participated in this meeting. Seventeen care units were included in the study, eight hospital care units (regional healthcare sector) and nine nursing home care units (municipal care sector). The care units appointed a team of three to seven members who would be responsible to implement and perform risk assessments using the TilThermometer at the unit. The team included the care unit manager and different HCWs, such as nurses, assistant nurses and physiotherapists. The purpose of having a team at each care unit was to ensure that they had the staff resources, available time and ability to manage the study and perform the intervention with the TilThermometer.

### Risk assessment with the instrument TilThermometer

**Fig. 1:**
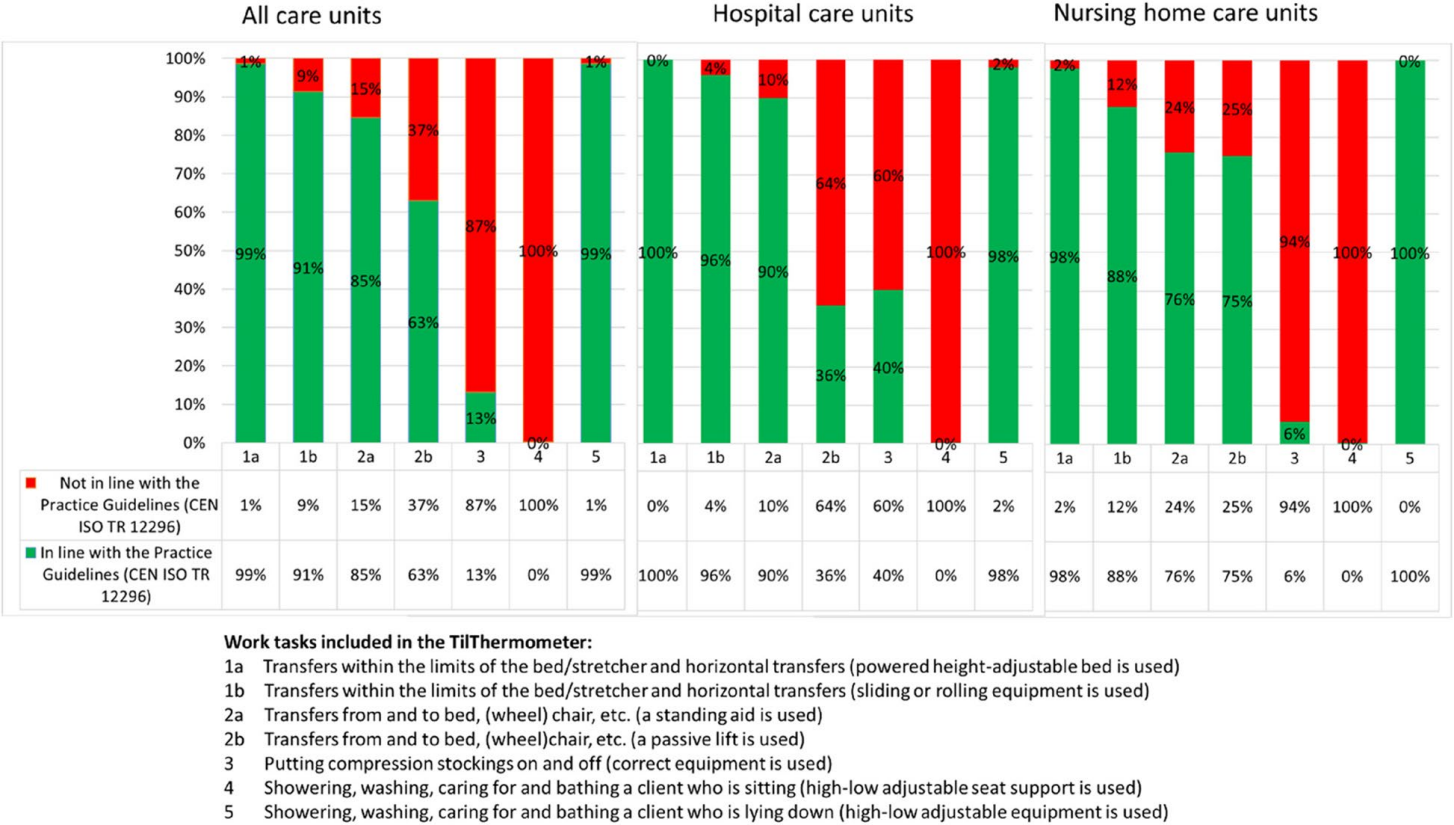
Presentation of the risk profile for physical exposure generated by using the TilThermometer

Risk assessment with TilThermometer, part 1; The risk assessment of the HCWs’ exposure to risk of high physical load when working with PHM is performed in relation to the care recipients’ function and level of activity [22–24]. Five different work task with transfers and caring situations are assessed and used as the basis for summarizing a risk profile: 1. Transfers within the limits of the bed/stretcher and horizontal transfers; 2. Transfers from and to bed, (wheel) chair; 3. Putting on and taking off compression stockings; 4. Showering, washing, caring for and bathing a client who is sitting; 5. Showering, washing, caring for and bathing a client who is lying down (Fig. 1).

For work task 1 and 2, five different mobility groups A-E, are used to classify the mobility of care recipients in relation the work tasks described above. In mobility group A, the care recipient can carry out the movement by him or herself with or without assistive devices, whereas in mobility group E, the care recipient is completely passive and needs total support and assistance from the HCWs. The five mobility groups are presented in table in Table 1 and on the official webpage of the TilThermometer, available and free to use [24].

**Table 1:** Definition of the mobility groups A-E

**Mobility group**	**Definition**
A	Client can independently perform the action, with or without equipment, and without risk of physical overload. Stimulating self-reliance is of great importance.
B	Client needs help, but this does not cause physical overload. Stimulation self-reliance is of great importance.
C	Client needs help. This may cause physical overload for the health care workers when equipment is not used. The client can however substantially contribute to the action. Stimulation activity and self-reliance is of great importance.
D	Client needs help. This causes physical overload for the health care workers when equipment is not used. Client is very passive and can barely contribute. Stimulation activity and self-reliance remain of great importance.
E	Client needs a lot of help. This causes physical overload for the health care workers when equipment is not used. Client is completely passive. Stimulating activity is not a priority: comfort and safety are the main concern.

For work tasks 3-5, the care recipient need for support from HCWs is observed and documented as either yes or no. Therefore, no mobility group classification is assigned.

Risk assessment with TilThermometer, part 2; After assessing mobility groups for work tasks 1 and 2 and evaluating the requirement for support from HCWs in work tasks 3-5, the TilThermometer incorporates an assessment regarding whether assistive devices/aids are utilised in each PHM situation for each care recipient. This assessment is conducted for all five PHM work tasks. [22, 23]. The risk assessment with the TilThermometer is first based on the risk assessment of each individual care recipient. Then, the results from all care recipients at the unit are aggregated and summarized into a risk profile for physical strain in HCWs at that care unit. The aggregated result is presented as follows: for work tasks 1 and 2 the number of classified patients in mobility groups C-E and for work tasks 3-5 the number of patients in need of support from HCWs. Additionally, for all five work tasks, it is documented whether assistive devices have been used for the care recipients presented above (green for used, red for not used). The results are presented with percentages and in a histogram with colours (Fig. 1).

### Study procedures and the use of TilThermometer

In order to standardize the implementation of the TilThermometer an introduction, a video film on *How to use the TilThermomete*r was sent to the teams in all units. To further support the participating care units, each unit was appointed a coordinator. The coordinators were ergonomists/physiotherapists, trade union representatives or healthcare developers. They had an active role of supporting the performance and documentation of the risk assessment along with the team at each care unit. A participatory approach was used to engage and involve the HCWs to perform the risk assessment with the TilThermometer at their care unit in a standardized manner. The coordinator’s participation was based on the need of support at each care unit. During a four-to-six-week period between September and November 2021, each team performed four risk assessments with the TilThermometer at their unit with at least one week apart. The risk assessments were carried out by the team in dialogue with the coordinator at each care unit, and thereafter documented. The TilThermometer was provided for use via the official webpage for the TilThermometer [24], where the risk assessment was documented and later saved as a PDF. The results of the risk assessment could be used directly by each care unit for their own work environment management.

### Data collection and outcome measures

Background data on participating care units were collected from the manager (i.e. type of organization, number of employees and care recipients). Demographic data were collected from the participants (gender, profession/role, part-time or fulltime work).To investigate if the TilThermometer can be used to assess risks for physical exposure in HCWs working with PHM and generate risk profiles, documented risk assessments with the Swedish version of the TilThermometer were collected from participating care units. Experiences of performing risk assessment with the TilThermometer were collected from activity logs, filled in by the team after each one of the four assessments. To assess the acceptability, appropriateness and feasibility of the TilThermometer, three short, validated implementation outcome measures were used [27, 28], complemented by eight research specific questions on perceived usefulness. For details on these measures see paragraph 2.5.3. The questionnaires were web-based and sent to the managers, team members and coordinator at all participating units when all four assessments had been made (November 2021).

### Risk assessment and identification of risk profiles

The teams conducted and documented in total four risk assessments using the TilThermometer at their unit. The results from each individual care recipient were compiled at group level, providing an overall summary of a risk profile for physical exposure in HCWs at the care unit. Risk profiles for physical exposure were summarized for each participating hospital and nursing home care unit. The risk profiles were related to the practice guidelines based on ISO/TR 12996:2012 [29], where green results indicates being in line with the guidelines and red not being in line.

### Experiences of using the TilThermometer

Qualitative data on experiences of performing risk assessments using the TilThermometer were collected by an activity log, filled in by the care units’ manager, the coordinator and/or a team member after performing each of the four risk assessments. The activity log included open ended questions on what worked well and what difficulties were encountered when using the TilThermometer.

#### Acceptability, appropriateness, feasibility, and usefulness of the TilThermomete

The following implementation outcome measures were used [27, 28]; the Acceptability of Intervention Measure (AIM), the Intervention Appropriateness Measure (IAM), and the Feasibility of Intervention Measure (FIM) [27, 28]. These “leading indicators” of implementation success [30] are validated questionnaires with the purpose of assessing the fit and match of an intervention, in this study risk assessment with the TilThermometer, in a given context targeting different criteria [28]. The questionnaires comprise four items each, answered on 5-point Likert-type scales: (1) Strongly disagree; (2) Disagree; (3) Neither agree nor disagree; (4) Agree; (5) Strongly agree. In general, higher scores tend to indicate a more positive perception or acceptance of the implementation object, whereas lower scores may suggest areas requiring attention or improvement. To further evaluate the usefulness of risk assessment with the TilThermometer, eight research-specific questions were added: Risk assessment with the TilThermometer 1) clarifies what is important connected to the transferring of the care recipient; 2) reduces unnecessary workloads; 3) gives management a basis for planning daily care at the unit; 4) increases the HCWs’ opportunity to participate in decision-making; 5) gives HCWs better feedback from risk assessments; 6) gives HCWs a chance to deal with problems before they become serious; 7) working with risk assessments fits well with the unit’s needs and working methods; and 8) in my unit, I want us to continue working with risk assessment to promote safe PHM. The answers were given on a 5-point Likert-type scale: 1) Strongly disagree; (2) Disagree; (3) Neither agree nor disagree; (4) Agree; (5) Strongly agree. These research specific questions regarding the measurement of usefulness were created with inspiration from a previous study by Sandqvist et al. [31] where the evaluation of an instrument's usefulness was assessed.

### Data analysis

Descriptive data are presented with mean and SD, median and range, or counts and percentages. Comparisons between hospital care units and nursing home care units were analysed using independent sample t tests. The limit for statistical significance was set at <0.05. The quantitative data were analyzed using the IBM SPSS version 25. The qualitative data were analyzed with qualitative content analysis using an inductive approach [32], by experienced qualitative researchers. The text in the activity logs were read several times to provide a comprehensive picture. Meaning units describing both positive and negative experiences were coded by one of the authors (ENS), and grouped into sub-categories and main categories based on similarities and differences. Consensus discussions on categories were held with two other authors (CW, PE). All three authors agreed on the categories.

## Results

The main results of this feasibility study are presented in four sections; 3.1 Risk profile for physical exposure; 3.2 Available assistive devices and equipment; 3.3 Experiences of using the TilThermometer and; 3.4 Acceptability, appropriateness, and feasibility of the TilThermometer. The data were collected from 17 care units, including eight hospital care units and nine nursing home care units. In total, 54 HCWs participated in this study. The majority were assistant nurses (52%) followed by registered nurses (11%), physiotherapists/ergonomists (9%), organizational developers (15%), and managers (13%). All participants were involved in a team at their healthcare unit. Most HCWs were women (94%) and the vast majority (90%) worked more than 75% or full-time.

### Risk profiles for physical exposure

The risk assessments with the TilThermometer were based on a total of 747 care recipients, 54% in hospital care units and 46% in nursing home care units. In work task 1, transfers within the limits of the bed/stretcher and horizontal transfers, 31% (*n*=231) of the care recipients were assessed in the TilThermometer mobility groups C, D and E (Table 2). For work task 2, 32% (*n*=242) of the care recipients were assessed in mobility groups C, D and E.

**Table 2:** Number and percentage of care recipients in hospital and nursing home care units classified into the mobility groups C, D and E according to the TilThermometer work tasks 1 and 2. For work tasks 3-5, the care recipients in need of support for each PHM^a^ are presented in percentages

	All care units	Hospital care units (Regional)	Nursing home care units (Municipal)
Total number (%) of care recipients	747 (100%)	405 (54%)	342 (46%)
1. Transfers within the limits of the bed/stretcher and horizontal transfers (Mobility groups C, D, E)	231 (31%)	101 (25%)	130 (38%)
2. Transfers from and to bed, (wheel) chair, etc. (Mobility groups C, D, E)	242 (32%)	109 (27 %)	133 (39%)
3. Putting on/taking off compression stockings	114 (15%)	25 (6%)	89 (26%)
4. Showering, washing, caring for and bathing a care recipient who is sitting	427 (57%)	150 (37%)	277 (81%)
5. Showering, washing, caring for and bathing a care recipient who is lying down	77 (10%)	50 (14%)	19 (6%)

^a^*PHM* Patient Handling and Movement

The risk assessments generated risk profiles for physical exposure at the units. The profiles were different for care units in hospitals and nursing homes (Fig. 1).

Risk for physical exposure was reported as high in two work tasks; showering sitting down (work task 4) and putting compression stockings on (work task 3), Fig. 1. Support from HCWs was needed for 57% (n=427) of the care recipients concerning showering, washing, caring for and bathing when sitting. No care unit used a high-low adjustable seat when showering care recipients sitting down (work task 4). A total of 15% (*n*=114) of the care recipients needed support from HCWs putting on and taking off compression stockings (work task 3), and 13% used the recommended equipment. Correct equipment was more frequently used in hospital care units (40%) compared to care units in nursing homes (6%). In total, 10% (*n*=77) of the care recipients were provided with support when showering, washing, caring for and bathing lying down (work task 5). When showering, washing, caring for and bathing a care recipient who is lying down, high-low adjustable equipment was used in 99% of these situations. When performing transfers from and to bed/chair (work task 2a), standing lifts were more often used in care units in nursing homes (76%) compared to hospital care units (36%).

### Available assistive devices and equipment

**Fig. 2:**
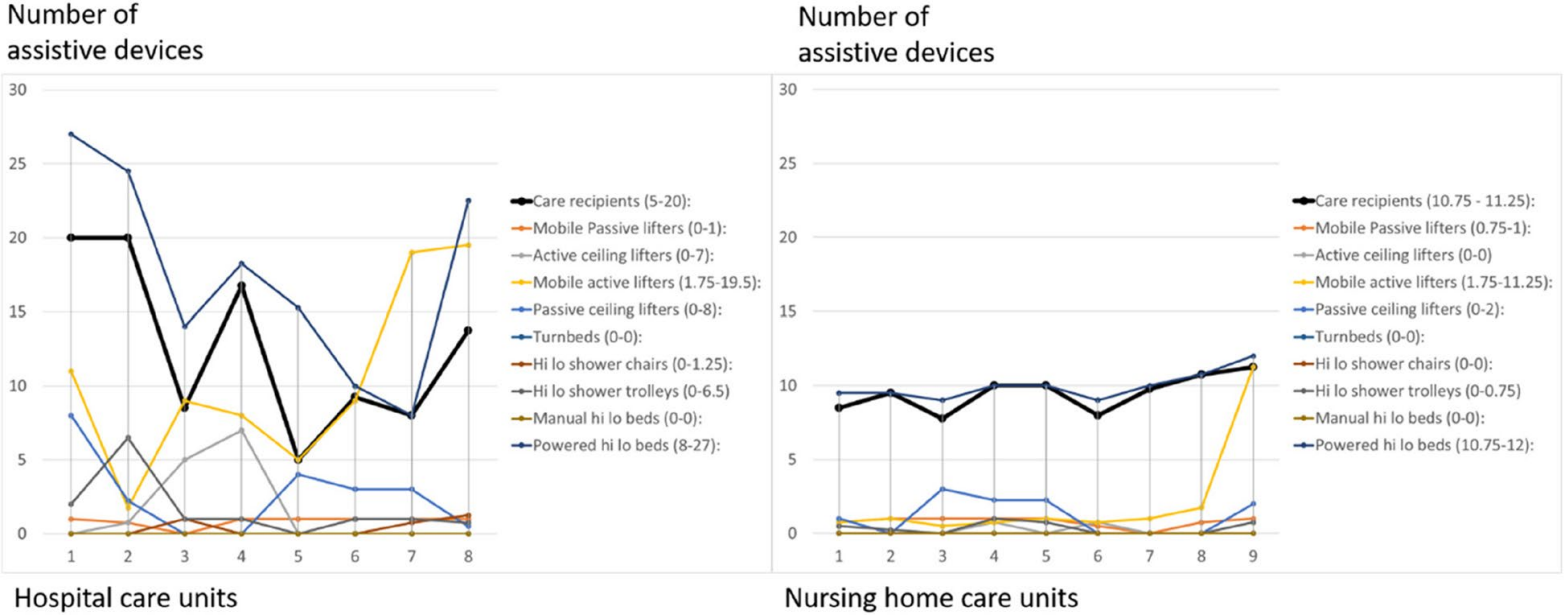
Number of care recipients, available assistive devices and equipment at eight hospital care units and nine nursing home care units

Slide sheets were the most frequently used assistive device; however, the availability of slide sheets ranged from 1 to 50 per care unit. The mean value for hospital care units was 22.3 and for nursing home care units, 4.75. Other assistive devices available at the units are presented in Fig. 2.

### Experiences of using the TilThermometer

The analysis of the activity logs, where the HCWs documented their experiences of using the TilThermometer, generated two overarching categories: “Facilitators of performing risk assessment with the TilThermometer” and “Difficulties when using the TilThermometer”.

#### Facilitators of performing risk assessment with the TilThermometer

Overall, the HCWs described the TilThermometer as easy to use. It was easy to fill in the data in the system and easy to grip. However, categorization of care recipients and distinguishing between mobility groups required practice. Risk assessments with the TilThermometer enabled reflection over working tasks and workload, as well as over care of recipients’ physical functioning and need for assistive devices. Reflections and team discussions on each patient and their assistance needs were highly valued and warranted as they were rare in usual practice. The TilThermometer provided an overview of care recipients’ need for assistance, use of assistive devices, and an overview of the unit’s workload. The HCWs emphasized that a risk assessment with TilThermometer needs to be made by at least two HCWs together, both knowing the care recipient. The risk assessment was facilitated by thinking through the unit’s care recipients, with the description of mobility groups in mind or as a fact sheet.

#### Difficulties when using the TilThermometer

Most of the described difficulties concerned uncertainty about how to categorize each care recipient into the five different mobility groups. It was especially difficult to distinguish between the mobility groups C and D and between D and E, and to interpret what “may need help” means. The statements revealed, however, a learning process where the categorization into mobility groups became easier over time when more risk assessments had been completed, and the HCWs had learned to use the TilThermometer. The uncertainty in categorization in mobility groups could also be because of challenges in the assessment of care recipient’s functioning and mobility due to variations in functioning over the day, when the patient is new at the unit, has cognitive impairments, or lacks motivation. Care recipients receiving palliative care or being immobilized due to medical reasons were also difficult to categorise and it was questioned whether the instrument was appropriate for acute surgery and palliative care units. Furthermore, there was uncertainty about what should be included in the assistive devices, primarily regarding adjustable bed/stretcher, compression stockings and aids used for rehabilitation (walking aid, tilting table/standing bed). The need for assistive devices like adjustable shower chair or bed, patient lifts, varied depending on whether the assessment was made in washing while sitting or while showering.

#### Acceptability, appropriateness and feasibility of the TilThermometer

The AIM/IAM/FIM questionnaires provided data on the HCWs’ perspective of implementation outcomes. Acceptability (AIM), which measures whether risk assessment using TilThermometer was agreeable, scored a total mean of 3.93 (SD 0.65). For appropriateness (IAM), measuring the perceived relevance and fit of the risk assessment, the total mean was 3.99 (0.66) (Table 2). The total mean for feasibility (FIM), the extent to which TilThermometer could be implemented and used by the HCWs, was 3.97 (0.73) (Table 3). None of the participants chose the strongly disagree option. When comparing the results for regional hospital care units with nursing home care units, there was a significant difference concerning the three items of AIM, where the nursing home units scored significantly higher. The same pattern was shown for IAM and FIM (Table 3).

**Table 3:** Acceptability, appropriateness, and feasibility of using the TilThermometer to assess risks for physical exposure. Comparison between hospital and nursing home care units

**Construct and item**	**All care units** **Mean (SD)**	**Hospital care units** **Mean (SD)**	**Nursing home** **care units Mean (SD)**	***P*-Value**
Acceptability (AIM) (*n*=54)*				
This risk assessment is pretty good	3.9 (0.60)	3.8 (0.63)	4.2 (0.40)	0.005**
This risk assessment is appealing	3.9 (0.63)	3.8 (0.65)	4.3 (0.45)	0.007**
I like this risk assessment	3.9 (0.65)	3.7 (0.64)	4.2 (0.54)	0.013*
I welcome use of this risk assessment	4.0 (0.71)	3.9 (0.70)	4.3 (0.68)	0.073
Total mean value (AIM)	3.93 (0.65)			
Appropriateness (IAM) (*n*=54)*				
This risk assessment seems fitting	3.8 (0.65)	3.7 (0.66)	4.1 (0.50)	0.021**
This risk assessment seems suitable	3.8 (0.61)	3.7 (0.62)	4.2 (0.40)	0.004**
This risk assessment seems applicable	4.0 (0.70)	3.9 (0.74)	4.2 (0.54)	0.087
This risk assessment seems well aligned	3.8 (0.68)	3.6 (0.67)	4.2 (0.54)	**0.003**
Total mean value (IAM)	3.9 (0.66)			
Feasibility (FIM) (*n*=54)*				
This risk assessment seems implementable	3.9 (0.68)	3.8 (0.75)	4.2 (0.40)	0.091
This risk assessment seems workable	4.0 (0.66)	3.9 (0.69)	4.3 (0.48)	0.015**
This risk assessment seems doable	4.0 (0.66)	3.9 (0.69)	4.3 (0.48)	0.015**
This risk assessment seems easy to use	3.9 (0.77)	3.8 (0.79)	4.2 (0.66)	0.049*
Total mean value (FIM)	**3.97 (0.73)**	3.8 (0.75)	4.2 (0.4)	0.045*

All items’ responses are measured on a 5-point Likert-type scale from (1) Strongly disagree to (5) Strongly agree. A higher score indicates a better outcome on all the three measures: the Acceptability of Intervention Measure (AIM), the Intervention Appropriateness Measure (IAM) and the Feasibility of Intervention Measure (FIM) [28]. * *p*<0.05, ** *p*<0.005

#### Usefulness of the risk assessment

The HCWs’ responses to the eight research specific questions evaluating usefulness of risk assessment with the TilThermometer are presented in Table 4. The score was reported significantly higher among HCWs in nursing home units when comparing two of the statements: doing risk assessment with the TilThermometer clarifies what is important in connection with transferring a care recipient, 4.4 mean value versus 4.1 mean value with (p-value: 0.027), and give healthcare workers a chance to deal with problems before they become serious, 4.4 mean value versus 4.2 (*p*-value: 0.030).

**Table 4:** Healthcare workers’ responses to eight statements concerning usefulness of TilThermometer

**Item Doing risk assessment with the TilThermometer**	**Total Mean (SD)**	**Hospital care units Mean (SD)**	**Nursing home care units Mean (SD)**	***P*-Value**
clarifies what is important connected to the transferring of care recipient	4.1 (0.71)	4.0 (0.75)	4.4 (0.50)	0.027*
reduces unnecessary work loads	3.9 (0.67)	3,8 (0.68)	4.1 (0.62)	0.066
gives management more basis for planning of daily care at the unit	4.0 (0.70)	3.9 (0.69)	4.3 (0.68)	0.093
increases the healthcare workers’ opportunity to participate in decision-making	4.1 (0.64)	4.0 (0.66)	4.3 (0.58)	0.173
gives healthcare workers better feedback from risk assessments	4.1 (0.64)	4.1 (0.73)	4.1 (0.34)	0.708
gives healthcare workers a chance to deal with problems before they become serious	4.2 (0.67)	4.1 (0.70)	4.4 (0.51)	0.030*
fits well with the unit’s needs and working methods	4.1 (0.66)	4.1 (0.69)	4.1 (0.62)	0.918
In my unit, I want us to continue working on risk assessment to promote safe patient handling and movements	4.2 (0.75)	4.1 (0.78)	4.3 (0.68)	0.581

All items’ responses are measured on a 5-point Likert-type scale. Scale ranging from strongly disagree (1) to strongly agree (5) A higher score indicates a better outcome on each item. *p<0.05

## Discussion

In this feasibility study, we evaluated acceptability, suitability and the ability to implement the Swedish version of the risk assessment instrument TilThermometer in 17 care units, including eight hospital care units and nine nursing home care units. In total, 54 HCWs participated in this study.

### Summary of findings

The TilThermometer was found to be useful for assessing risk for physical exposure in HCWs when working with PHM in a Swedish context. By first assessing each care recipient and then compiling the results at a group level, a summary of mobility groups and an overall risk profile for the HCWs. Risk assessment with the TilThermometer may help to identify the need for the proper assistive device. The study shows that the TilThermometer was perceived as both suitable and acceptable to the participants. The acceptability, appropriateness, feasibility, and perceived usefulness of performing risk assessments with the TilThermometer was reported as high among HCWs. The risk assessment enabled reflection about working tasks related to PHM, workload, and about care recipients’ physical functioning and the need for assistive devices.

### Risk assessment with the TilThermometer

The Swedish version of the TilThermometer can be used both to map the care recipients’ mobility level as well as HCWs’ exposure to physical load in relation to PHM in hospital and nursing home care units. The highest reported work task of risk for high physical exposure was when showering a care recipient sitting down and when putting on compression stockings. The risk profiles differed between settings, where for an example standing lifts were more often used in nursing home care 4 units compared to hospital care units. The study shows promising results regarding the TilThermometers’ ability to assess care recipients into different mobility groups, in a Swedish context. One-third of the care recipients were categorized in the TilThermometer mobility groups C, D and E, which means that many of the care recipients’ had limited mobility and needed help to various degrees from HCWs with the transfer. When the mobility and health of a care recipient is limited, and they are older, there is also increased risk of falls [2, 33]. Patients with partial dependency on transfer was found to have a significantly higher risk of falls than independent patients [33]. Similar risk factors for falls were found among older people living in nursing homes, such as impaired ADL performance, fall history, poor balance and depression [34]. Acknowledging the significance of mobility during hospitalization, alongside recognizing the adverse impacts of immobility, is a crucial initial stride towards developing effective interventions promoting mobility and independence [35]. The care recipients’ health and functional status is also directly related to the HCWs' workload [36]. Patient-related factors such as older age, several diagnoses, low functional status, and being unable to self-care, are some of the identified risk factors for increased HCW workload [36]. Greater functional independence and higher mobility levels in patients have shown to reduce staff levels of care [37]. The TilThermometer can provide a profile for each care unit concerning patients’ mobility levels as well as risk profiles for physical exposure, which can be useful when determining the need for staff and provision of required equipment.

### Availability and use of assistive devices and equipment

Risk assessment with the TilThermometer may help to identify the connection between the patients’ functional ability in relation to the use of the proper assistive device. Findings from previous research shows that the availability of equipment, annual training and supervisor encouragement is associated with increased use of equipment by HCWs [10]. This study shows that the TilThermometer can be used at Swedish care units to map HCWs’ use of available assistive devices and equipment in various PHM situations. Overall, the most used assistive devices were sliding sheets and standing aids, whereas the compression stocking applicators were not available and therefore less commonly used. The scarce use of compression stocking applicators is a concern, as the applicators can significantly lower frequency of forces exerted [38]. WMSDs in shoulder/arm and hand are common among HCWs [4, 7]. The work task of assisting care recipients in putting on compression stockings is very physical demanding, which could potentially explain some of the prevalence. In general, the importance of using assistive devices to decrease the physical workload and reduce the risk of WMSDs has been emphasised in previous research [11, 12, 14, 39]. Bathing a patient is acknowledged as a physically demanding work tasks [40]. One findings in the present study was that none of the care units used electric high-low adjustable seats when showering care recipient sitting down. In a study by Neval et al. [41] muscular activity of the back muscles and the perceived musculoskeletal strain after work were lowered with use of the electrically adjustable shower chair compared to using a traditional shower chair. On the other hand, almost all care units used high-low adjustable equipment when showering, washing, caring for and bathing a care recipient who is lying down. Also, powered height-adjustable beds are frequently used in all care units. Both adjustable bed and shower chairs are suggested to decrease the duration of awkward back postures for HCWs [38]. In Sweden, as in many countries, the managers are responsible for working with systematic work environment management and perform risk assessments and to provide work equipment necessary for promoting safe PHM [16].

### HCWs’ experiences of using the TilThermometer

How the end-users, those delivering or receiving an intervention, perceive the features of the intervention is a factor that is known to strongly affect implementation outcomes [42, 43]. Successful implementation depends largely on the fit of evidence-based interventions with the preferences and priorities of those who shape, deliver, and participate in care [44]. In this study, the AIM/IAM/FIM questionnaires [28] provided information on the HWCs’ perspectives of implementation outcomes, and an opportunity for them to retrospectively assess the feasibility of the risk assessment instrument, TilThermometer. This evaluation of the fit and match showed that the HCWs scored high on all three outcomes, implying that the HCWs believed that the TilThermometer is acceptable, appropriate, and feasible for assessing risks in PHM situations at their healthcare units. The HCWs judged the TilThermometer as feasible, as it could be used for risk assessments relatively easily or conveniently given existing resources and circumstances at the unit. As previously suggested, assessing these outcomes early in the research process may ensure that intervention is optimized and fits with end-users’ preferences [28]. Recently, a small feasibility study explored the implementation of a risk assessment program for patient handling in a long term care setting in Australia [45]. In line with the findings in the present study, the staff reported that the program was acceptable and practical to implement, but there was a need to practise the program regularly [45]. Taken together, these findings indicate that staff in various healthcare settings welcome the implementation of interventions for safe PHM, but they may need support and training. There are several risk assessment instruments that can be used when assessing exposure during PHM. Villaroya et al. [20] made a comparison based on items selected from five risk assessment instruments. At present, only The Direct Nurse Observation Instrument (DINO) and TilThermometer is officially translated to Swedish [24, 46]. The support from an ergonomist is recommended when performing the DINO method, but the TilThermometer could be used by HCWs themselves. There are today no previous descriptions of how the end-users perceive risk assessment with any of these instruments. The HCWs in the present study described risk assessment using the TilThermometer as easy and useful, especially after being acquainted with the instrument. The risk assessment instrument enabled them to reflect on care recipients’ functioning and their need for assistive devices, and provided an overview of HCWs’ physical load related to PHM at the unit. However, there were also descriptions of difficulties in assessing care recipients’ functioning, and categorization in some of the mobility groups, and the value of the TilThermometer in acute settings was questioned. As previously been proposed in implementation frameworks, intervention features such as trialability (ability to test the intervention in a small scale) and complexity of the intervention, promote successful implementation of the intervention [47, 48]. Thus, the findings in this study indicate that risk assessment using the TilThermometer could be adopted by HCWs at Swedish care units in hospitals and nursing homes. Further research is also needed to investigate if interventions including risk assessment promotes safe PHM and reduce musculoskeletal injuries and disorders among HCWs [21]. In the present study the TilThermometer was implemented in a small scale providing knowledge on the use of the TilThermometer for a larger cluster randomized trial in the Swedish healthcare sector [49].

### Strengths and limitations

One of the aims of performing this study was to clarify any uncertainty about the feasibility of implementing the risk assessment instrument TilThermometer in a Swedish healthcare setting, and to use the findings to design a future randomized controlled trial. Conducting pilot or feasibility studies before main studies is proposed to reduce research waste [50] and is important for increasing the chances of successful delivery of the future study [51]. Collecting data through the TilThermometer website enabled easy access to unbiased data. Other strengths are the use of validated questionnaires to assess acceptability, appropriateness, and feasibility [28], and the structured activity log to collect experiences of using the TilThermometer, analyzed with qualitative content analysis [32] by experienced qualitative researchers. A study limitation might be that the focus was only on those HCWs receiving the intervention and the use of study-specific questions evaluating the usefulness of the risk assessment with the TilThermometer. The sampling strategy reached a variation of settings and distribution of healthcare professionals. However, the sampling strategy was convenient to reach interested care units in hospitals and nursing homes. This could imply that we included care units with higher awareness of occupational safety and health and interested in improving the safety regarding PHM.

### Clinical implication

The systematic assessment of risks in the work environment is the first step to deciding which measures are relevant. The use of the risk assessment instrument TilThermometer was found to be a feasible approach to assess risk of physical exposure among HCWs in relation to PHM. A team-based way of working was used to implement the risk assessment with TilThermometer. Our findings show the importance of being a team of HCWs, where discussion can be held in relation to the risk assessment, both on how to use it and for reflecting on the findings. This can be an active part of promoting the safety culture at the care units. This study provides new knowledge about if and how the TilThermometer can be used in a Swedish healthcare setting. The TilThermometer is free and available to use, and is described in the Swedish evidence-based guideline for safe patient handling published in 2022 [39].

## Conclusion

The findings from this feasibility study suggest that the TilThermometer can be used to assess risks for physical exposure in relation to patient handling and movement in hospital and nursing home care units. The risk assessment provided risk profiles with potential to contribute to care units’ development of a safe patient handling and movement practice. The healthcare workers’ experiences of risk assessment using the TilThermometer indicate that interventions for safe patient handling and movement incorporating the instrument may be adopted by healthcare workers at Swedish hospitals and nursing home care units.

## Data Availability

The data that support the findings of this study are available on request from the corresponding author (C.W.).

## Abbreviations

PHM: Patient handling and movement
HCWs: Healthcare workers
WMSDs: Work-related Musculoskeletal disorders
